# Detection of clinically silent brain lesions in [18F]FDG PET/CT study in oncological patients: analysis of over 10,000 studies

**DOI:** 10.1038/s41598-021-98004-w

**Published:** 2021-09-14

**Authors:** Agata Pietrzak, Andrzej Marszałek, Jolanta Kunikowska, Tomasz Piotrowski, Adrianna Medak, Katarzyna Pietrasz, Julia Wojtowicz, Witold Cholewiński

**Affiliations:** 1 Electroradiology Department, Poznan University of Medical Sciences, Garbary 15, 61-866 Poznan, Poland; 2 Nuclear Medicine Department, Greater Poland Cancer Centre, Garbary 15, 61-866 Poznan, Poland; 3 Oncologic Pathology and Prophylaxis Poznan University of Medical Sciences and Greater Poland Cancer Center, Garbary 15, 61-866 Poznan, Poland; 4 Nuclear Medicine Department, Medical University of Warsaw, Banacha 1A, 02-097 Warsaw, Poland; 5 Medical Physics Department, Greater Poland Cancer Centre, Garbary 15, 61-866 Poznan, Poland; 6 Medical Faculty, Łodz Medical University, Tadeusza Kościuszki 4, 90-419 Lodz, Poland

**Keywords:** Cancer, Medical research, Molecular medicine, Oncology

## Abstract

The study aimed to show that including the brain region into the standard 2-deoxy-2-[18F]fluoro-d-glucose positron emission tomography/computed tomography ([18F]FDG PET/CT) study protocol may result in detecting clinically silent brain tumours. We retrospectively analyzed the group of 10,378 from the total of 12,011 consecutive patients who underwent the torso and brain [18F]FDG PET/CT scanning, considering an ability of the method to evaluate undetected before brain tumours in patients diagnosed and treated in our institution. While collecting the database, we followed the inclusion criteria: at least 1-year of follow-up, a full medical history collected in our institution, histopathologic examination or other studies available to confirm the type of observed lesion, and the most importantly—no brain lesions reported in the patients’ medical data. In this study, performing the torso and brain [18F]FDG PET/CT imaging helped to detect clinically silent primary and metastatic brain tumours in 129 patients, and the benign lesions in 24 studied cases, in whom no suspicious brain findings were reported prior to the examination. In conclusion, including the brain region into the standard [18F]FDG PET/CT protocol can be considered helpful in detecting clinically silent malignant and benign brain tumours.

## Introduction

The worldwide incidence of the primary brain tumours approximates at less than 2% of all detected tumours. The most common are the metastatic brain foci^1–3^ and the benign lesions. Although, most of the benign lesions are not lethal, they often cause several health ailments, including neurological or locomotory disorders caused by the tumour’s growth. Therefore, in some cases, the benign brain lesions undergo surgical resection. Malignant brain tumours are often deadly and demand therapy. The method of choice in the brain tumours treatment is the surgical resection of the malignant foci^1–3^. However, surgery is not always available due to tumour’s location, the number of detected brain lesions (i.e. oligometastatic brain disease), or the patient’s poor general health condition. Currently, the malignant brain tumours remain difficult to detect and their therapy is often unsuccessful. Moreover, the lesion’s location and a high risk of a post-surgical complications among the brain tumour patients limit the possibility to perform the lesion’s histologic evaluation^2–5^. Thus, the diagnostic imaging seems essential in the brain tumours patients management. The methods used in the brain tumours diagnosis are the contrast-enhanced and non-enhanced Magnetic Resonance Imaging (ceMRI, MRI), contrast-enhanced computed tomography (ceCT) and the positron emission tomography/computed tomography (PET/CT)^6^. The most used PET-dedicated radiotracer in oncology is the 2-deoxy-2-[18F]fluoro-d-glucose ([18F]FDG). However, a high physiologic glucose uptake in the cortex limits the usefulness of the [18F]FDG in evaluating brain foci^7–9^. Thus, in some of the nuclear medicine departments, the standard [18F]FDG PET/CT acquisition protocol does not include the brain region scanning^10,11^. Depending on the clinical indications, the [18F]FDG PET/CT acquisition protocol can be performed as the whole-body, torso, limited-area tumour imaging, and lastly, the whole-body or torso imaging combined with dedicated brain imaging^12^. The area of the scanning usually ranges from the base of the skull to the mid-thigh. Presented in this research study protocol including the area from the skull-apex to mid-thigh can be described as the torso and brain^18^FDG PET/CT imaging.

Detecting both the benign and malignant brain tumour may be considered vital for the patient’s outcome, and can be often considered life-saving. In this study, we presented the data supporting the hypothesis that including the brain region into the daily [18F]FDG PET/CT procedure performance can provide the valuable data regarding the developing brain pathology despite the commonly recognized study limitations.

This study aims to show that performing the torso and brain [18F]FDG PET/CT scanning may help to incidentally detect clinically silent brain tumours.

## Results

### Brain findings—overview

We analyzed 12,011 consecutive patients who underwent the torso and brain [18F]FDG PET/CT examination in our institution. We included in the final analysis the total of 10,378 patients. In this group, we found malignant brain tumours in 129 patients, and benign lesions in 24 of studied subjects. According to patients’ medical records, the most of evaluated brain lesions have been surgically resected and histopathologically examined. In case of a high post-surgical mortality risk or the oligometastatic brain disease detected, causing histopathological evaluation impossible to conduct, we used the diagnostics imaging techniques (MRI, ceCT, follow-up [18F]FDG PET/CT studies) to analyze brain findings.

We found 24 primary brain tumours, 1 to 3 metastatic brain foci in 105 patients (the total of 137 brain metastases obtained), and 24 cases of the benign brain lesions occurrence. To ensure the collected results reliability, we included in the analysis only one tumour obtained in each metastatic brain tumour patient.

We analyzed the medical records of the 10,378 patients to evaluate the ability of the torso and brain [18F]FDG PET/CT method to detect the brain tumour despite the commonly recognized method’s limitation. In this group, we found 129 malignant and 24 benign brain lesions. Accordingly with the patients’ medical data, no brain lesions have been reported in the remaining 10,225 group of patients, examined with the torso and brain [18F]FDG PET/CT acquisition protocol.

### Primary brain tumours

24 oncological patients (14 women, 10 men, mean age ± standard deviation; S.D.: 63 ± 13 years old (y.o.), age range: 26–84 y.o.) underwent the [18F]FDG PET/CT study due to the following clinical indications to perform the study: cancer of unknown primary (CUP; 16), colorectal cancer (2—rec. ass.), breast cancer (1—recurrence assessment, rec. ass.), esophageal cancer (1—rec. ass.), lung cancer (1), uteri cancer (1—rec. ass.), prostate cancer (1), melanoma malignant (1). Some of the examined patients have been treated before. However, the brain lesions have not been reported prior to the torso and brain scanning.

**Figure 1:**
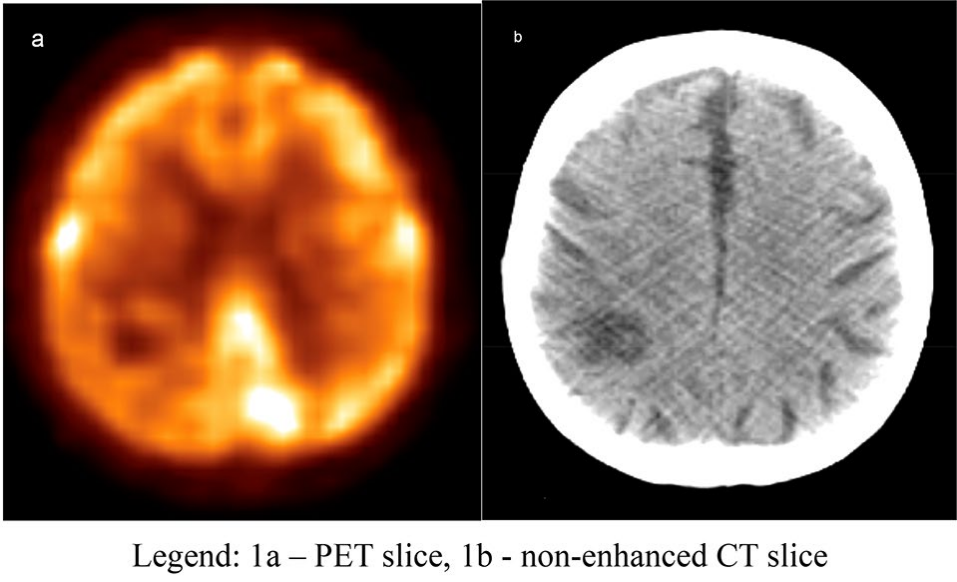
54 y.o. patient scanned with the [18F]FDG PET/CT (CUP syndrome). Abnormal finding within the right parietal lobe showing a lesion with decreased [18F]FDG uptake surrounded by the brain tissue oedema (*source*: original figure; software—Philips Fusion Viewer). (**a**) PET slice, (**b**) non-enhanced CT slice.

In 19 from 24 of studied patients, single brain lesion was detected with the [18F]FDG PET/CT method, and in 5 cases the brain tumour was one of a multiple evaluated malignant lesions due to advanced stage of the primary disease (i.e. lymph nodes involvement, metastatic bone tumours; Fig. 1).

Figure 1 shows the primary brain tumour discovered in CUP syndrome patient.

### Brain metastases

We found the total of 137 metastatic brain tumours in 105 patients (up to 3 foci in one patient). We excluded from the final analysis 32 metastatic foci, using the total tumour’s volume and the highest [18F]FDG uptake value as the inclusion criteria. The studied group consisted of 65 women, 40 men (mean age ± S.D.: 59 ± 12 y.o., age range: 30–81 y.o.), scanned with the torso and brain [18F]FDG PET/CT method to evaluate the breast cancer in 27 cases (22—staging, 5—restaging with one patients diagnosed of both breast and lung cancer), melanoma malignant (25), lung cancer (20 patients in whom 3 diagnosed of both lung and colorectal cancer), colorectal cancer (9), non-Hodgkin’s lymphoma (4), ovarian cancer (4), prostate cancer (3), gastrointestinal cancer (3; 1—staging, 2—restaging), renal cancer (3), CUP (2), thyroid cancer (2), uteri cancer (2), pancreatic tumour (1).

**Figure 2:**
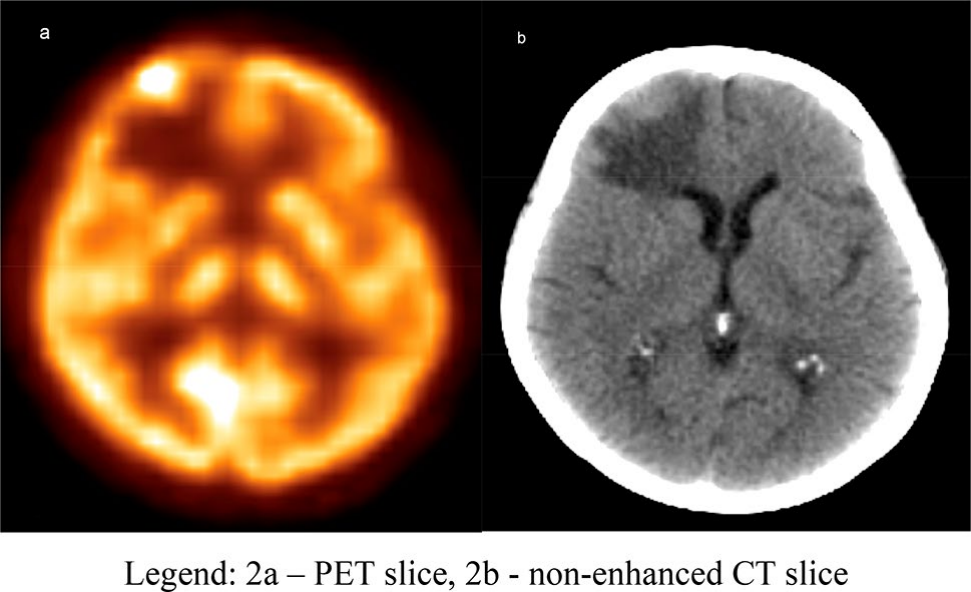
46 y.o. patient with ovarian cancer scanned with the [18F]FDG PET/CT before surgery. Abnormal finding within the right frontal lobe showing a lesion with increased [18F]FDG uptake surrounded by brain tissue oedema—ovarian cancer metastasis (*source*: original figure; software—Philips Fusion Viewer). (**a**) PET slice, (**b**) non-enhanced CT slice.

The metastatic brain tumour was the only one evaluated malignant lesion in 39 from 105 patients (37.1%), and one of a few detected tumours in the patient’s body in 13 cases (12.4%). In 50.5% of the analyzed scans, we obtained the advanced oligometastatic disease with the significant lymph nodes involvement and the secondary bone tumours observed (Fig. 2).

Figure 2 shows the metastatic brain lesion, obtained with the torso and brain [18F]FDG PET/CT study.

### Benign brain lesions

We found the benign brain lesions in 24 patients (15.7% of all detected lesions). Based on patient’s follow-up data (medical records including diagnostic imaging and histopathologic examination), the benign lesions group included 21 arachnoid cysts, 2 meningiomas and 1 adenoma in 16 women, 8 men (mean age ± S.D.: 63 ± 13 y.o., age range: 35–84 y.o.). Patients underwent [18F]FDG PET/CT scanning to evaluate the treatment effectiveness in colorectal (6), breast (5), cervical (4), ovarian (2), melanoma malignant (2), CUP (2), renal (1), testicular (1), prostate (1) patients.

Accordingly with the patients’ medical records lesions did not demand therapy. However, detecting brain foci resulted in recommending a regular follow-up studies due to the risk of the possible future side-effects caused by the tumour’s growth.

### Glucose metabolism activity of the brain lesions

We measured glucose metabolism activity within the analyzed groups of brain lesions, using PET-dedicated parameter of the maximal standardized uptake value (SUVmax). According to the Shapiro–Wilk test’s results, the SUVmax value distribution within the groups of the primary, metastatic and the benign lesions was Gaussian with *P* = 0.9, *P* = 0.7, *P* = 0.1, respectively (Table 1). Among the evaluated brain tumours, we found both [18F]FDG—avid and non—[18F]FDG—avid lesions.

**Table 1:** The SUVmax calculations: benign, primary and metastatic brain tumours ( source: original data).

Groups/characteristics	SUVmax^a^ value ± S.D.	SUVmax Median	SUVmax value range	CI_95_^b^
**Value**				
All brain lesions	8.3 ± 2.6	8.7	1.7–14.7	[7.9; 8.7]
Primary brain tumours	9.1 ± 1.3	9.3	6.5–12.0	[8.7; 9.7]
Brain metastases	9.1 ± 2.0	9.1	4.1–15.0	[8.7; 9.5]
Benign lesions	4.2 ± 1.8	3.9	1.7–9.0	[3.4; 5.0]

*S.D.* standard deviation.

^a^SUVmax—maximal standardized uptake value.

^b^CI_95_—95% confidence interval (valid for at least 95% of studied population considering the SUVmax value mean).

Table 1 shows the measurements.

According to the Student t-test’s for the independent groups of variables, the benign and malignant lesions’ metabolic activity differed significantly with *P* < 0.001. According to the Kruskal–Wallis test’s results, the SUVmax value difference between the primary and metastatic brain lesions was insignificant (*P* = 0.9). The SUVmax value dataset obtained within the benign lesions group differed significantly from the malignant tumours groups (*P* = 0.02).

### Benign and malignant brain lesions differential diagnosis

The [18F]FDG PET/CT method is a widely used imaging technique for the oncological purposes, among which the especially important seems to be distinguishing between the benign and malignant tumours in a various regions of the human body. According to the literature^13^, the SUVmax value exceeding 2.5 should be considered abnormal. However, a homogenously high [18F]FDG uptake within the grey matter results in observing the increased SUVmax value levels within the brain. Therefore, the possibility to detect a small brain lesion and to perform the differential diagnosis seems difficult using the SUVmax value of 2.5 criterion. In this study, we performed the Receiver Operating Characteristics (ROC) analysis to calculate the SUVmax value cut-off which may differentiate between the benign and the primary brain lesions in this database. The evaluation of cut-off points distinguishing all benign and all malignant tumours, and the primary and metastatic brain tumours was omitted due to significant differences in sample-sizes between those groups of malignant foci.

**Figure 3:**
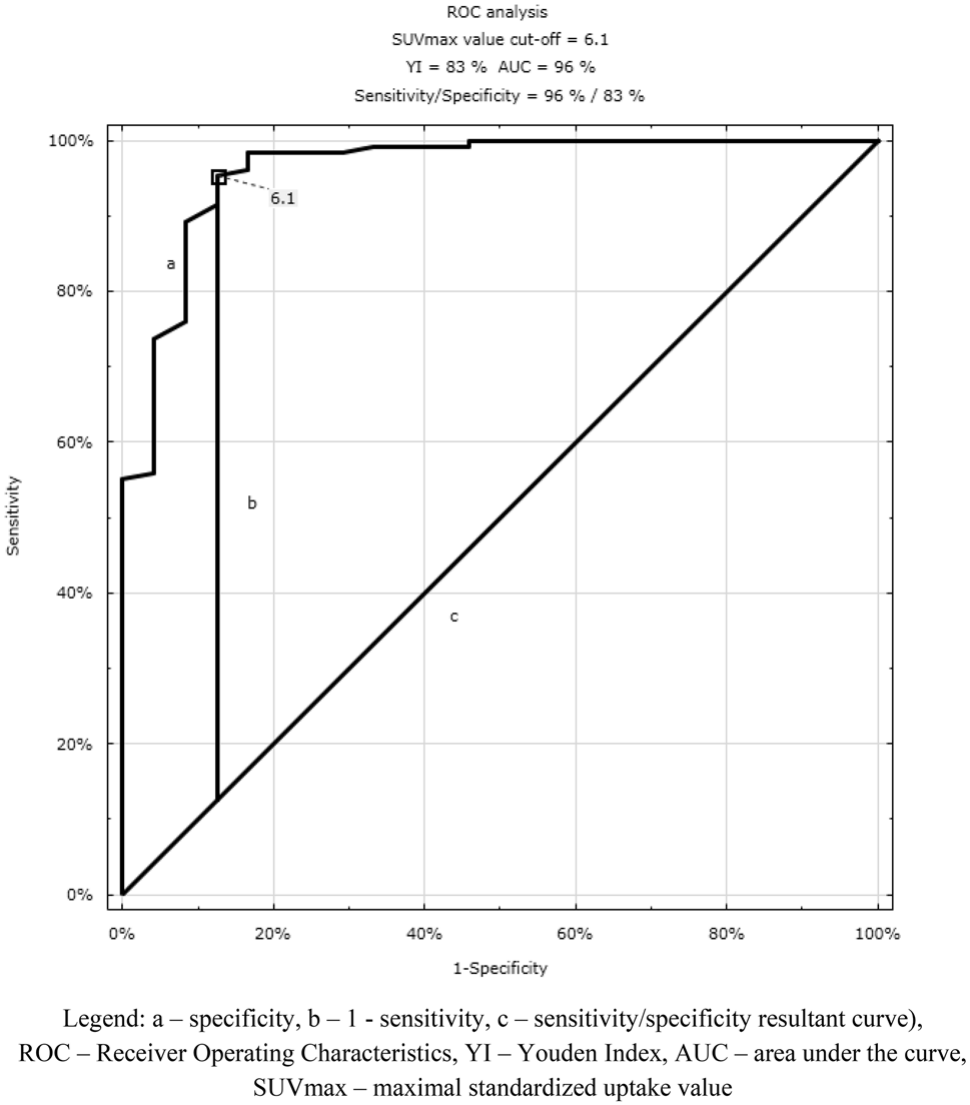
SUVmax value cut-off: benign and malignant brain lesion ( *source*: original figure, STATISTICA, Statsoft). (**a**) specificity, (**b**) 1—sensitivity, (**c**) sensitivity/specificity resultant curve), *ROC* Receiver Operating Characteristics, *YI* Youden Index, *AUC* area under the curve, *SUVmax* maximal standardized uptake value.

When compared the SUVmax value levels between groups of the benign and primary brain lesions, we found the SUVmax value cut-off suggesting the malignant pathology was 6.1 with the sensitivity and the specificity of the method of 96%, 83%, respectively (Fig. 3).

## Discussion

The study aimed to indicate the importance of the incidental brain lesions detection in the oncological patients, using the torso and brain [18F]FDG PET/CT acquisition. Although, the study is not considered a method of choice in the brain diagnosis, it remains one of the most used and accessible oncological imaging techniques. Discovering the primary or secondary brain tumours seems to be crucial due to high risk of the lethal effect of a developing malignant brain lesion as well as due to several neurological and cognitive ailments observed in patients suffering from the brain tumours. Authors^12,14–16^ discussed the applications and limitations of the torso and brain [18F]FDG PET/CT study, indicating a low sensitivity and the specificity of the method in detecting small tumours located within the brain. In some of the nuclear medicine departments performing the PET/CT scanning using the [18F]FDG might be the only way to support their oncological patients due to the limited accessibility to the more suitable for the brain tumours diagnosis radiopharmaceuticals, i.e. tumours’ hypoxia or increased cells’ proliferation indicators^17,18^.

In this study, we detected clinically silent brain tumours in 153 from examined 10,378 oncological patients (1.5% of examined population), among which nearly 90% occurred to be malignant, and 24 were benign. The available follow-up data of the remaining 10,225 studied subjects did not mention any other cases of the brain tumour being diagnosed. Detecting benign brain pathologies might not effect with the necessity of the surgical resection of the obtained foci, and according to the available medical records, none of the evaluated 24 benign lesions underwent surgical resection. However, the discovery demands follow-up studies to avoid possible side-effects connected with the tumour’s growth, including the neurological, locomotory, and cognitive disorders. Moreover, in some cases, the developing benign brain lesion may also be lethal due to, i.e., progressing destruction of the brain tissue caused by the increasing over time lesion’s volume. In this database, in every condition, the regular follow-up studies were recommended.

Most often, malignant brain foci undergo treatment as deadly. The patients in whom the malignant brain foci was detected are monitored more carefully, especially if the primary or metastatic brain focus is the only one or one of a few detected symptoms of the developing oncological disease. According to the patients’ medical records, all of 129 malignant brain tumours patients received treatment (surgical resection, chemotherapy, surgery combined with radiotherapy; depending on the staging of the disease, tumour’s location, patients’ general health condition).

We have included into the final analysis the patients’ in whom the brain lesion were not reported previously in any other available studies. In some of cases, the histopathologic data were not available due to high post-surgical risk of mortality. In this group, we used other imaging methods’ results and the follow-up studies. To ensure a full access to the patients’ medical records, we included in the study patients diagnosed and treated only in our institution (patients’ transferred to other hospitals to continue the therapy were excluded due to insufficient follow-up data).

According to authors^12,16^, the torso and brain [18F]FDG PET/CT study can be considered useful in the benign and malignant brain tumours differential diagnosis. In this study, we used the ROC analysis to evaluate the SUVmax value cut-off, distinguishing benign and primary lesions. The SUVmax value cut-off differentiating between malignant and benign brain lesions was 6.1 with a considerably high sensitivity and specificity of the method (96%, 83%, respectively).

The oncological patients’ database is heterogenous due to institution characteristics (wide range of examined diagnoses) which may be considered the limitation of the method. Moreover, the differences in the sample-sizes between the groups of the benign, primary and metastatic brain tumours limited the possibility to establish the SUVmax value cut-off, distinguishing the benign and metastatic, and the primary and metastatic tumours. Finally, the relevance of the obtained results may be considered subjective and depending on the clinician’s individual assessment whether to use the torso and brain [18F]FDG PET/CT in the daily practice.

In this study, performing the torso and brain [18F]FDG PET/CT examination resulted in the incidental detection of the clinically silent brain tumours, and gave 129 patients a chance to successfully undergo the oncological therapy, which would not be possible if the 1 min of the brain scanning would be excluded from the [18F]FDG PET/CT acquisition protocol. Thus, inclusion of the brain region into the standard acquisition PET/CT protocol might be worthy consideration.

## Methods

### Bioethics

The study is based on the retrospective anonymized analysis approved by the Local Bioethical Committee (Poznan University of Medical Sciences, prof. Pawel Checinski, date of approval: 30.01.2020). This study includes the original studies performed upon patients’ informed consent in writing due to the standard institution protocol. The study is based on the unsponsored, single-institutional studies, using the database collected in 2011 to 2019. All data have been anonymized and the examined patients cannot be identified. All steps of the examinations have been performed in accordance with the Bioethical Committee guidelines and the Declaration of Helsinki.

### Statistical analysis

In this study, we performed the comparative analysis of the PET-dedicated parameter of SUVmax, obtained withing the groups of the benign brain lesions, primary brain tumours, and the metastatic brain foci. Prior the final analysis, we used the Shapiro–Wilk normality test and divided datasets into independent groups of lesions. We followed the statistical significance level of *α* = 0.05 (confidence interval at the level of 95%, CI_95_), and summarized the statistical tests’ results, considering *P* value. When analyzed the results, we used a null and alternative hypotheses (H_0_, H_a_, respectively) assumptions: H_0_ suggested that the true variables’ distribution was normal or the evaluated differences between the calculations were statistically insignificant (*P* > 0.05), H_a_—true distribution significantly differed from Gaussian or the observed differences were significant (*P* < 0.05)^19^. In this study, we used the following statistical tests: the Kruskal–Wallis’, and the Student t-tests for independent variables. Furthermore, we performed the ROC analysis to establish the predictive SUVmax value cut-off distinguishing the benign lesions and the primary brain tumours glucose metabolism activity in this database (including the YI, and AUC parameters).

We used the STATISTICA, StatSoft software, version 13.3 (TIBCO Software, Palo Alto, California, USA, available upon individual license).

### Database collection

In this study, we have retrospectively analyzed 12,011 consecutive patients examined with the [18F]FDG PET/CT technique in our institution. In this group, we collected the database consisting of 10,378 patients, evaluated using the torso and brain [18F]FDG PET/CT^12^ studies. We included into analysis the total of 10,378 studies, following the criteria shown in the Table 2.

**Table 2:** 10,378 studies collection conditions: inclusion and exclusion criteria ( source: original data).

10,378 torso and brain [18F]FDG PET/CT studies database collection conditions	
Inclusion criteria	Exclusion criteria
Torso and brain [18F]FDG PET/CT study protocol performed in our institution to ensure a full access to the images, ranging from the skull-apex to mid-thigh	Other than torso and brain imaging [18F]FDG PET/CT (dated protocols omitting the brain region, a low quality of the brain scanning, incomplete brain imaging, movement or other artefacts observed)
The patients’ medical records available (including the therapeutic management data)	Incomplete medical records (including: studies performed in other hospitals, treatment continued in external institution)
At least 1-year^a^ of the patients’ follow-up: the brain lesion’s type confirmed using the histopathological examination (depending on the possibility to perform surgery) or other studies (ceCT, MRI, repeated torso and brain [18F]FDG PET/CT imaging)	Insufficient follow-up, no other than torso and brain [18F]FDG PET/CT study available to confirm the lesion’s type or to analyze the further patients’ management
No brain lesion reported in the patients’ medical records	Brain tumour previously detected or suspected prior to the [18F]FDG PET/CT study
Clinically silent brain lesion observed	Symptoms of the developing brain lesion reported (i.e. neurological or cognitive ailments)

^a^Range: 1–4 years of follow-up.

### Study performance

The torso and brain [18F]FDG PET/CT study has been performed at 60 min (min).

post-injection (p.i.) of the radiopharmaceutical [18F]FDG in the mean activity of 337 ± 69 megabecquerels (MBq), range: 152–544 MBq (administered activity up to 3.7 MBq/kg of body mass). The [18F]FDG PET/CT study has been performed using the Philips Gemini TF16 hybrid scanner (Philips, Cleveland, Ohio, United States of America, USA).

The acquisition protocol included the area of skull-apex to mid-thigh (patients laid supine with arms above the head)^12^. PET imaging preceded Body Low-Dose CT using the following parameters: 150–245 miliamperseconds (mAs), 120–140 kilovoltage peak (kVp), Pitch of 0.8. PET section scanning time was 90 s (s). The scanning duration did not exceed 35 min (min), including 1 min of the brain region imaging. To evaluate lesions within the brain, we chose the semi-automatic method of contouring, using the Philips-dedicated software Fusion Viewer (Philips, Cleveland, Ohio, USA).

## Data Availability

The datasets analyzed during the current study are available from the corresponding author on request.
